# Validating the association of Oxford classification and renal function deterioration among Taiwanese individuals with Immunoglobulin A nephropathy

**DOI:** 10.1038/s41598-023-49331-7

**Published:** 2023-12-11

**Authors:** Cheng-Hsu Chen, Ming-Ju Wu, Shang-Feng Tsai

**Affiliations:** 1 Division of Nephrology, Department of Internal Medicine, Taichung Veterans General Hospital, 160, Sec. 3, Taiwan Boulevard, Taichung, 407 Taiwan; 2 Department of Life Science, Tunghai University, Taichung, Taiwan; 3 Department of Post-Baccalaureate Medicine, College of Medicine, National Chung Hsing University, Taichung, Taiwan; 4 Ph. D. Program in Tissue Engineering and Regenerative Medicine, College of Medicine, National Chung Hsing University, Taichung, Taiwan

**Keywords:** Medical research, Nephrology

## Abstract

Validation of the Oxford classification (MEST and MEST-C) for Immunoglobulin A nephropathy (IgAN) in the Taiwanese population is lacking. Our study aimed to validate this classification and assess individual lesion impact. We conducted a retrospective cohort study at Taichung Veterans General Hospital, Taiwan (Jan 2011–Jul 2023). Composite renal outcomes were evaluated using clinical conditions and estimated glomerular filtration rate (eGFR). We used Kaplan–Meier, univariable/multivariable logistic regression and ROC curves. Subgroup analysis considered eGFR < or ≥ 30.0 ml/min/1.73 m^2^. In 366 renal biopsies, serum creatinine was 1.34 mg/dl, eGFR 53.8 ml/min/1.73 m^2^, urine protein–creatinine ratio 1159 mg/g. T1/T2 lesions had lowest baseline eGFR (39.6/11.5 ml/min/1.73 m^2^), correlating with poorest renal survival (median survival 54.7/34.4 months). Univariable analysis linked all individual variables to worse renal outcomes. Multivariable analysis (MEST/MEST-C) showed only T1/T2 linked to worse outcomes. T score had highest predictive power (AUC 0.728, sensitivity 60.2%, specificity 83.6%), with MEST having high AUC (0.758). No extra predictive power was seen transitioning MEST to MEST-C. Subgroup analysis (eGFR < 30.0 ml/min/1.73 m^2^) associated C1 with improved renal outcomes (odds ratio 0.14, 95% CI 0.03–0.65). T lesion correlated with worse outcomes across subgroups. The T lesion consistently correlated with worse renal outcomes across all groups and baseline statuses. Integrating the C lesion into the transition from MEST to MEST-C did not enhance predictive power. Importantly, the C1 lesion was linked to improved renal outcomes in the eGFR < 30.0 ml/min/1.73 m^2^ subgroup, likely due to treatment effects.

## Introduction

Immunoglobulin A nephropathy (IgAN) is the main glomerulonephritis globally, especially in Asian populations^1^, including Taiwan (26.0% prevalence)^2^. Due to diverse outcomes, predicting renal results is vital. A past study^3^, affirmed Haas classification's efficacy in Taiwanese IgAN outcomes, using 2003–2013 data. Our goal now is to validate the global Oxford classification in our larger IgAN patient group. The Oxford classification (MEST) has been employed for predicting renal outcomes since 2009^4,5^ and has garnered validation across more than 20 cohorts^6–12^, including Europe, North America, Europe, Japan, South Korea and China. Notably, the Oxford classification has not been previously validated within the Taiwanese population. This validation is crucial to preempt any regional discrepancies in IgAN management. Once the Oxford classification is validated consistently across various countries, it can serve as a robust tool for predicting patient outcomes. Hence, it becomes essential to establish the predictive capability of the Oxford classification's outcomes in Taiwanese population. Given that our institute, Taichung Veterans General Hospital, handles the highest number of renal biopsy cases in Taiwan^13^, we believe that our study from this institution serves as a validation of the Oxford classification in Taiwan.

It should be noticed that the latest update from the 2021 Kidney Disease: Improving Global Outcomes (KDIGO) clinical practice guideline^14^ introduced a set of five variables (MEST-C) derived from pathology, encompassing mesangial hypercellularity (M), endocapillary hypercellularity (E), segmental glomerulosclerosis (S), tubular atrophy/interstitial fibrosis (T), and crescents (C). The primary reason is that the initial MEST did not include individuals with an eGFR < 30.0 ml/min/1.73 m^2^. After incorporating this population, the C lesion's association with renal function became apparent, leading to the revision of MEST to MEST-C^12^. However, not every study has the consistent results^9,10,15,16^. The validation of C score in other population should be performed. In our previous study^17^, we found that crescents IgAN had worse patient survival compared to non-crescents IgAN. However, the relevance of crescents to renal outcome in our specific population remains uncertain.

In addition, some previous validations lacked consistency due to their failure to achieve robust endpoints. As such, our study will adopt the most recent acceptable renal outcome criteria, as established in the 2020 international consensus^18^, which incorporates clinical parameters and glomerular filtration rate (GFR)-based laboratory outcomes. Given these factors, our study aims to validate the predictive capacity of the revised Oxford classification (MEST-C) in predicting renal outcomes specifically for Taiwanese IgAN patients.

## Materials and methods

### Definition of population

From January 2011 to July 2023, we undertook a retrospective cohort investigation at Taichung Veterans General Hospital located in Taiwan. This study encompassed individuals aged ≥ 20 years. To mitigate the impact of immunosuppression^19^, we select patients receiving their first renal biopsy (from January 2011 to July 2023), who are treatment-naive (not undergone any prior immunosuppressive treatment). The pathological reports of the renal biopsy were evaluated by two pathologists using the Oxford classification for IgA nephropathy. The final reports of the renal biopsy were concluded after reaching a consensus between these two pathologists. The research protocol was granted approval by the institutional review committee under the reference number CE15125B. All methods were performed in accordance with the relevant guidelines and regulations. The informed consent is waived due to de-identified data according to regulation of Institutional Review Board in our institute.

### Other data collection

Data was obtained from cohort medical records. Foundational information like gender, age (years), and BMI (kg/m^2^) were collected at biopsy. Blood data included serum creatinine (SCr) (mg/dl), estimated glomerular filtration rate (eGFR) (calculated by MDRD) (ml/min/1.73 m^2^)^20^, white blood cell count, hemoglobin (g/dl), platelet count, metabolic profile [total cholesterol (TC), triglycerides, LDL, HDL, fasting blood sugar (FBS), HbA1c (%), uric acid (UA)], and albumin (g/dl). Immunological markers were C3, C4, IgG, IgM, IgA, IgE. Urine samples assessed protein–creatinine ratio (UPCR) (mg/g), hematuria, pyuria. Pathologists diagnosed IgAN using revised Oxford classification (MEST-C).

### Outcome definitions

Initially, we linked variables to baseline renal outcomes (eGFR, UPCR). After long-term tracking, the study's endpoint aligned with 2020's global kidney failure criteria^18^. These definitions encompassed clinical criteria, such as instances involving kidney transplant, a minimum of 4 weeks' dialysis, instances where a participant passed away without initiating kidney replacement therapy, and situations where advanced chronic kidney disease was identified as the underlying cause of death. Furthermore, outcomes based on eGFR were taken into consideration. These included instances of doubling of SCr, a decline of at least 50% in eGFR from an initial baseline sustained over a minimum of 4 weeks, and eGFR levels below 15.0 ml/min/1.73 m^2^ sustained over at least 4 weeks. A similar definition of renal failure was also employed in a prior meta-analysis^8^.

### Statistical analyses

In this study, continuous variables were shown as median (Q1, Q3) for central tendency and data spread. Categorical data were expressed numerically (percentages) for frequency and proportion. Normality was checked using Kolmogorov–Smirnov Test. Chi-square tested categorical comparisons. Descriptive stats used Kruskal–Wallis (3 groups) and Mann–Whitney U (2 groups), as suitable.

Survival analysis used Kaplan–Meier (univariable) and Cox-proportional hazard analysis (univariable and multivariable) for renal survival curves. Log-rank test compared rates among risk groups. Univariable/multivariable logistic regression explored MEST-C's association with renal outcomes, providing odds ratios (ORs) and 95% confidence intervals (CIs). Logistic model fit was checked with Hosmer–Lemeshow test. Predictive capacity was assessed with ROC curves (AUC, sensitivity, and specificity), which was compared by DeLong test. Statistical analysis were performed with SPSS 22, and R 4.3.1. p value less than 0.05 was significant.

### Subgroup analysis

In a prior study conducted in Japan^21^, significant C lesion was associated with renal outcomes in baseline eGFR < 30.0 ml/min/1.73 m^2^ group. To gauge C lesion significance, we split cohort by baseline eGFR ≥ / < 30.0 ml/min/1.73 m^2^. Then, we analyzed with univariable/multivariable logistic regression and ROC curves in this subgroup.

### Ethics approval and informed consent to participate

Our study was approved by the Human Research Review Committee of the Taichung Veterans General Hospital (approval number CE15125B). All methods were performed in accordance with the relevant guidelines and regulations. The informed consent is waived due to de-identified data according to regulation of IRB in our institute.

## Results

### Baseline characteristics of Taiwanese IgAN cohort

Between January 2011 and July 2023, a total of 366 biopsies confirmed IgA nephropathy (IgAN) as detailed in Table 1. The median age within this cohort was 46.5 years. Notable observations revealed lower risks associated with metabolic syndrome: BMI at 23.5 kg/m^2^, fasting blood glucose (FBG) at 94 mg/dl, HbA1c at 5.5%, triglycerides at 122 mg/dl, LDL at 111.0 mg/dl, HDL at 47.4 mg/dl, and uric acid at 6.6 mg/dl. The incidence of comorbidities in this cohort was relatively low, including diabetes mellitus at 15%, coronary artery disease at 6.8%, arrhythmia at 2.7%, and congestive heart failure at 0.3%. Renal functions were compromised with serum creatinine (SCr) at 1.3 mg/dl, estimated glomerular filtration rate (eGFR) at 53.8 ml/min/1.73 m^2^, and urine protein-to-creatinine ratio (UPCR) at 1159.0 mg/g. Pathological diagnoses revealed findings of M in 58.4%, E in 29.7%, S in 62.3%, T in 29.2% (T1 in 20.4%, T2 in 8.8%), and C in 14.0% (C1 in 10.9%, C2 in 3.1%). A substantial majority of patients (80.8%) were on renin–angiotensin system inhibitors, while over half were prescribed pentoxifylline (54.6%) and statins (50.5%).

**Table 1:** Baseline characteristics for patients with IgA nephropathy, divided by with composite renal outcome or not.

Risk classification	Total (n = 366)	Without composite renal outcome (n = 253)	With composite renal outcome (n = 113)	p value
Variables	Median (Q1, Q3) for continuous variables; n (%) for categorical variables			
Demographic data				
Age (years old)	46.5 (37, 59)	46 (35, 59)	50 (40, 60)	0.004
Body mass index (kg/m^2^)	23.45 (20.70, 27.35)	24.00 (21.05, 27.78)	21.87 (20.00, 25.18)	0.004
Male	189 (51.6%)	136 (53.8%)	53 (46.9%)	0.258
Comorbidity				
Diabetes mellitus	55 (15%)	34 (13.4%)	21 (18.6%)	0.209
Coronary arterial disease	25 (6.8%)	15 (5.9%)	10 (8.8%)	0.37
Arrhythmia	10 (2.7%)	6 (2.4%)	4 (3.5%)	0.506
Congestive heart failure	1 (0.3%)	0 (0%)	1 (0.9%)	0.309
Medication				
Renin–angiotensin system inhibitors	295 (80.8%)	192 (76.2%)	103 (91.2%)	0.001
Pentoxifylline	200 (54.6%)	135 (53.4%)	65 (57.5%)	0.496
Dipyridamole	8 (2.2%)	6 (2.4%)	2 (1.8%)	1
Statin	185 (50.5%)	111 (43.9%)	74 (65.5%)	< 0.001
Blood data				
Blood WBC (/cumm)	7635 (6120, 9260)	7700 (6100, 9160)	7430 (6185, 10,010)	0.076
Hemoglobin (g/dl)	12.7 (10.75, 14.1)	13.2 (11.8, 14.4)	10.9 (9.1, 12.8)	< 0.001
Platelet (*10^3^/cumm)	247 (202, 292)	250 (209, 298)	224 (194, 270)	< 0.001
Fasting glucose (mg/dl)	94 (86, 107)	93 (86, 106)	96 (86, 109)	0.03
Glycated hemoglobin (%)	5.5 (5.3, 5.8)	5.5 (5.3, 5.8)	5.6 (5.2, 5.9)	0.123
Albumin (g/dl)	4 (3.6, 4.2)	4 (3.8, 4.3)	3.7 (3.3, 4)	< 0.001
Total cholesterol (mg/dl)	187 (164, 219)	186 (161, 219)	196 (170, 218)	0.395
Triglyceride (mg/dl)	122 (83, 186)	119 (82, 186)	135 (90, 184)	0.033
Low-density lipoprotein (mg/dl)	111 (91, 141)	107 (88, 139)	118 (92, 147)	0.427
High-density lipoprotein (mg/dl)	47.4 (31.2, 63.8)	47.6 (31.8, 63.4)	43.1 (29.7, 64.4)	0.604
Creatinine (mg/dl)	1.34 (0.9, 2.1)	1.1 (0.8, 1.57)	2.37 (1.62, 4.5)	< 0.001
Estimated glomerular filtrate rate (min/min. 1.732 m^2^)	53.83 (31.48, 86.11)	69.99 (44.7, 95.86)	26.39 (11.58, 42.23)	< 0.001
Uric acid (mg/dl)	6.6 (5.3, 8.19)	6.4 (5, 7.6)	7.5 (6.1, 9.19)	< 0.001
C3 (mg/dl)	111.6 (96.9, 126.4)	114.55 (101.8, 130.1)	99.3 (81.3, 118.5)	< 0.001
C4 (mg/dl)	30.65 (24.2, 37.1)	30.2 (23.7, 35.9)	32.29 (25.7, 38.29)	0.206
IgG (mg/dl)	1062 (899, 1268)	1112 (910, 1299)	1016 (811, 1197)	0.044
IgA (mg/dl)	327.65 (256.2, 417.9)	337.25 (258.8, 425.55)	304.85 (248.3, 373.5)	0.002
IgM (mg/dl)	97.7 (72.5, 131.95)	98.4 (74.2, 132.3)	92.9 (66.4, 129.1)	0.054
IgE (mg/dl)	69.3 (13.6, 95.3)	69.9 (30.4, 95.3)	4.7 (4.7, 4.7)	0.286
Urinary data				
Urine protein–creatinine-ratio (mg/g)	1159 (422.7, 2612.09)	870.53(340, 1660)	2070 (1040, 4240)	< 0.001
Hematuria	314 (85.8%)	208 (82.2%)	106 (93.8%)	0.003
Pyuria	208 (56.8%)	135 (53.4%)	73 (64.6%)	0.052
Pathological data based on Oxford classification				
M	206 (58.4%)	132 (52.8%)	74 (71.9%)	0.03
E	105 (29.7%)	62 (24.8%)	43 (41.7%)	0.012
S	220 (62.3%)	144 (57.6%)	76 (73.8%)	0.065
T1	72 (20.4%)	33 (13.2%)	39 (37.9%)	< 0.001
T2	31 (8.8%)	8 (3.2%)	23 (22.3%)	< 0.01
C1	39 (10.9%)	30 (12%)	9 (8.5%)	0.359
C2	11 (3.1%)	4 (1.6%)	7 (6.6%)	0.04

During the median follow-up of 36.5 months, 253 patients showed no renal complications, while 113 did. Notably, no missing data were identified. Patients exhibiting worse renal function were characterized by older age (50 vs. 46 years, p = 0.04), lower BMI (21.9 vs. 24.0 kg/m^2^, p = 0.04), decreased hemoglobin (10.9 vs. 13.2 g/dl, p < 0.001), reduced platelet count (224 vs. 250 K, p < 0.001), higher FBG (96.0 vs. 93.0, p = 0.03), lower serum albumin (3.7 vs. 4.0, p < 0.001), diminished triglycerides (119.0 vs. 135.0, p = 0.033), elevated SCr (2.4 vs. 1.1 mg/dl, p < 0.001), lowered eGFR (26.4 vs. 70.0 ml/min/1.73 m^2^, p < 0.001), reduced serum C3 (99.3 vs. 114.6 mg/dl, p < 0.001), decreased IgG (1016.0 vs. 1112.0 mg/dl, p = 0.044), and lower IgA (337.3 vs. 404.9, p = 0.002). Additionally, they exhibited higher UPCR (2070.0 vs. 871.0, p < 0.001) and increased hematuria (93.8 vs. 82.2%, p = 0.003).

Regarding histological findings, patients with the composite outcome demonstrated higher occurrences of M lesions (71.9 vs. 52.8%, p = 0.03), more frequent E findings (41.7 vs. 24.8%, p = 0.012), elevated T1 lesions (37.9 vs. 13.2%, p < 0.001), increased T2 lesions (22.3 vs. 3.2%, p < 0.001), and a higher prevalence of C2 lesions (6.6 vs. 1.5%, p = 0.04).

### Associations between pathological variables of MEST-C and baseline renal function (eGFR and UPCR)

Table 2 illustrates notable differences in individual pathology variables concerning baseline eGFR and UPCR. It was observed that lower baseline eGFR and higher UPCR were associated with more severe M, E, S, T, and C scores (all p < 0.05). Regarding baseline eGFR, notably lower values were observed for T1 and T2 (39.6 and 11.5 ml/min/1.73 m^2^, respectively), which were notably below the values of others (> 50.0 ml/min/1.73 m^2^). Concerning baseline UPCR, C1 and C2 displayed the highest values (2470.0 and 4705.0 mg/g, respectively), in contrast to the values observed in other categories, which were below 2400.0 mg/g.

**Table 2:** Correlations between pathological features and baseline renal outcomes (eGFR and UPCR).

	eGFR (ml/min/1.73 m^2^)	UPCR (mg/g)
	Median (Q1, Q3)	
Mesangial hypercellularity		
M0	69.97 (39.66, 94.53)	670 (260, 1565)
M1	47.2 (26.43, 75.58)	1510 (630, 3147.88)
p value	< 0.001	< 0.001
Endocapillary proliferation		
E0	61.08 (38.03, 91.86)	882.61 (322.1, 1701.5)
E1	42.56 (22.58, 71.15)	2377.81 (1166.47, 4560)
p value	< 0.001	< 0.001
Segmental glomerulosclerosis		
S0	65.24 (39.74, 95.86)	560 (200.55, 1542)
S1	50.19 (27.96, 82.55)	1472.43 (663.44, 3053.44)
p value	0.005	< 0.001
Tubular atrophy and interstitial fibrosis		
T0	71.46 (47.2, 96)	940.15 (340, 1790)
T1	30.58 (20.8, 44.25)	1843.5 (882.61, 3464.5)
T2	11.52 (7.36, 25.08)	2519 (1319, 5140)
p value	< 0.001	< 0.001
Cellular or fibrocellular cresent		
C0	56.85 (32.78, 87.11)	1035.5 (400, 1950)
C1	49.22 (32.18, 86.11)	2470 (1179.99, 3389)
C2	21.16 (10.63, 49.36)	4705.41 (2960, 9890)
p value	0.023	< 0.001

M, T, C by Kruskal–Wallis test.

E, S by Mann–Whitney U test.

### Renal survival among individual variables: M, E, S, T, and C lesions

**Figure 1:**
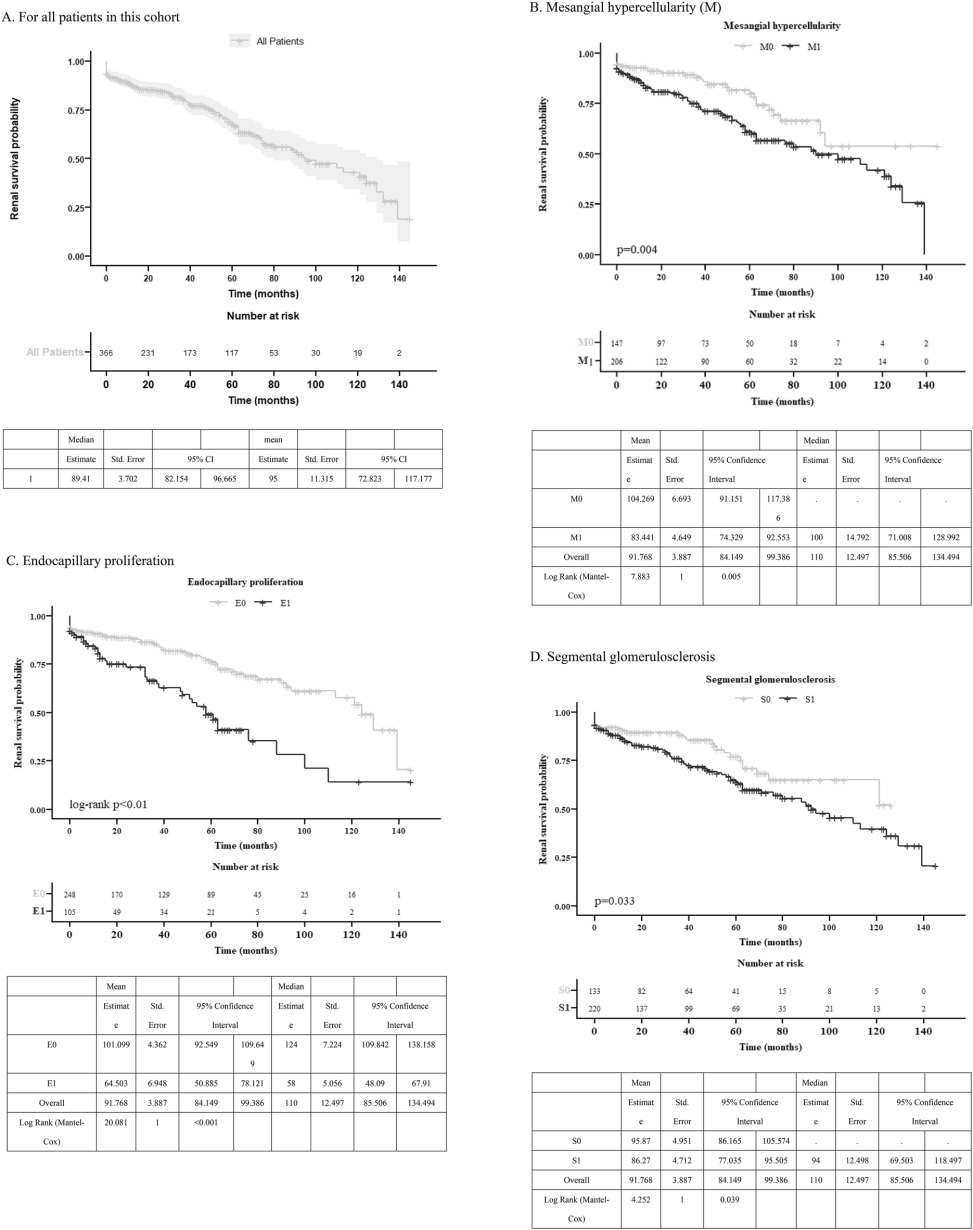

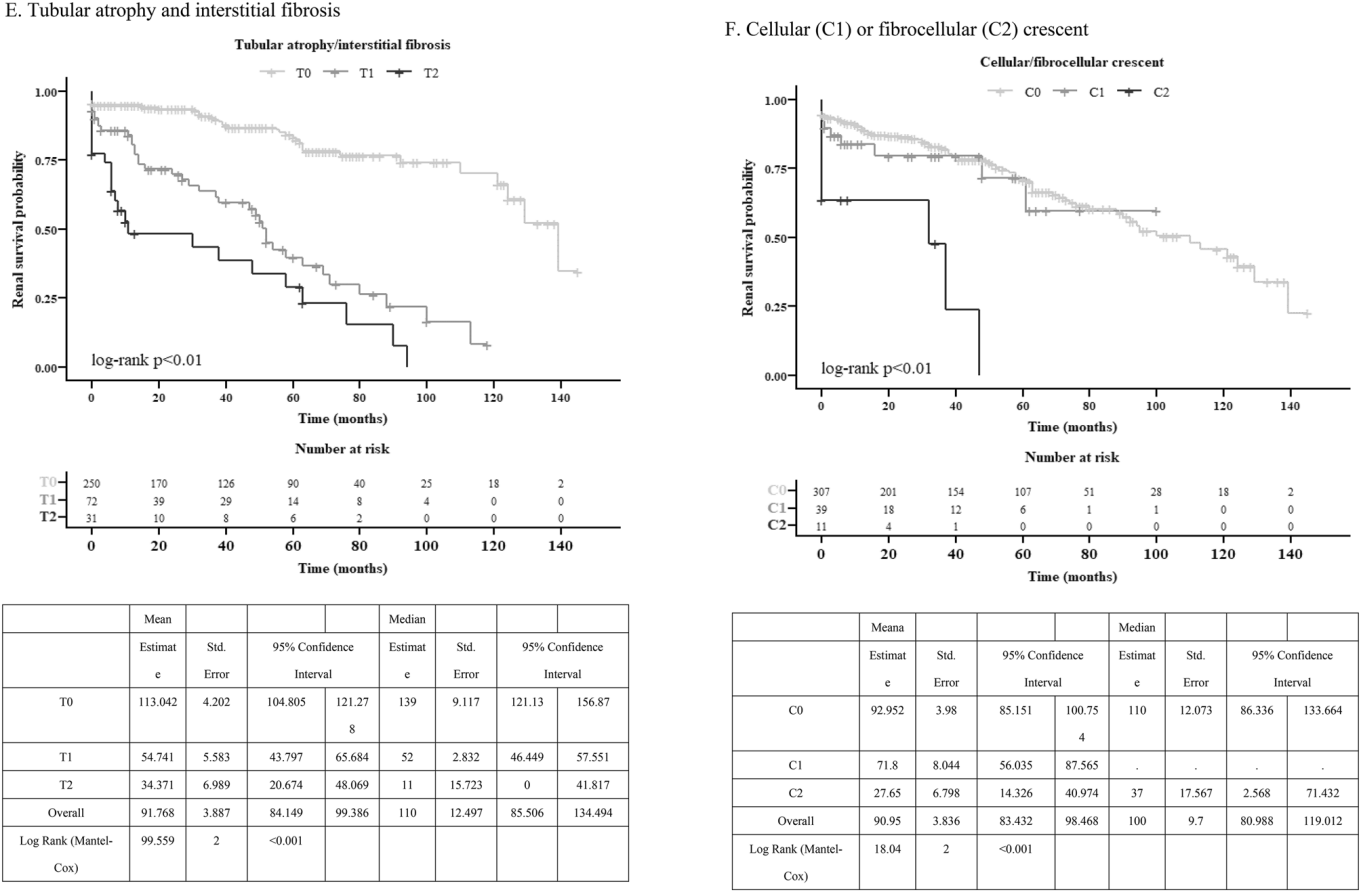
Kaplan–Meier survival curve of composite renal outcome. (**A**) For all patients in this entire cohort. (**B**) Mesangial hypercellularity. (**C**) Endocapillary proliferation. (**D**) Segmental glomerulosclerosis. (**E**) Tubular atrophy and interstitial fibrosis. (**F**) Cellular (C1) or fibrocellular (C2) crescent.

Over a 10-year follow-up, median survival of entire cohort was 89.4 months (Fig. 1A). Worse renal outcomes from survival curves can be noticed: M score (104.3 vs. 81.4 months, p < 0.005) (Fig. 1B), E score (101.1 vs. 64.5 months, p < 0.01) (Fig. 1C), S score (98.9 vs. 86.3 months, p = 0.033) (Fig. 1D), T score (113.4 vs. 54.7 vs. 34.4 months for T0 vs. T1 vs. T2, p < 0.01) (Fig. 1E), and C score (93.0 vs. 71.8 vs. 27.6 months for C0 vs. C1 vs. C2, p < 0.01) (Fig. 1F). Among all, T1 and T2 had poorest survival (54.7 and 34.4 months), followed by C1 and C2 (71.8 and 27.7 months).

### Associations between pathological variables of MEST-C and composite renal outcomes

**Figure 2:**
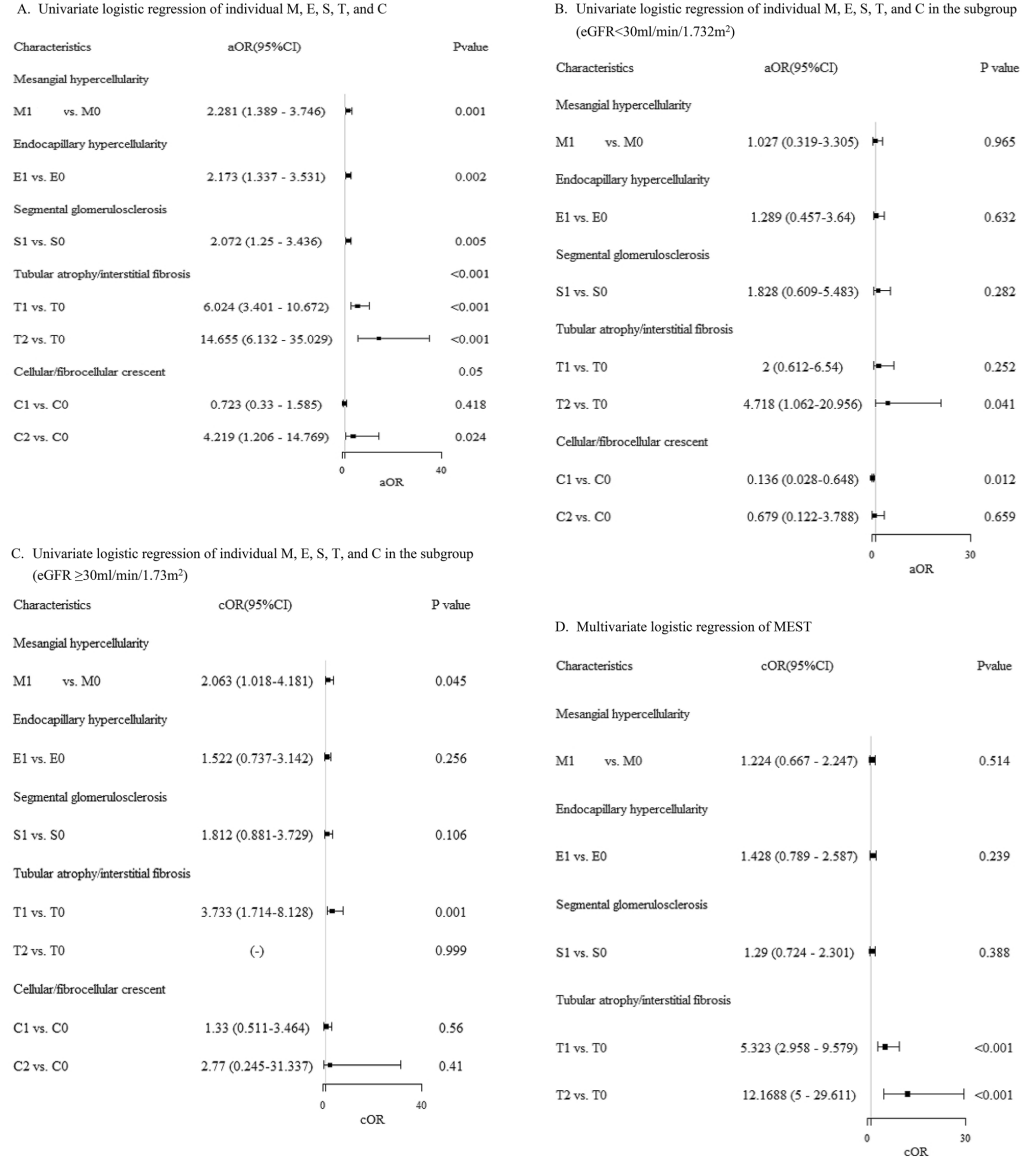

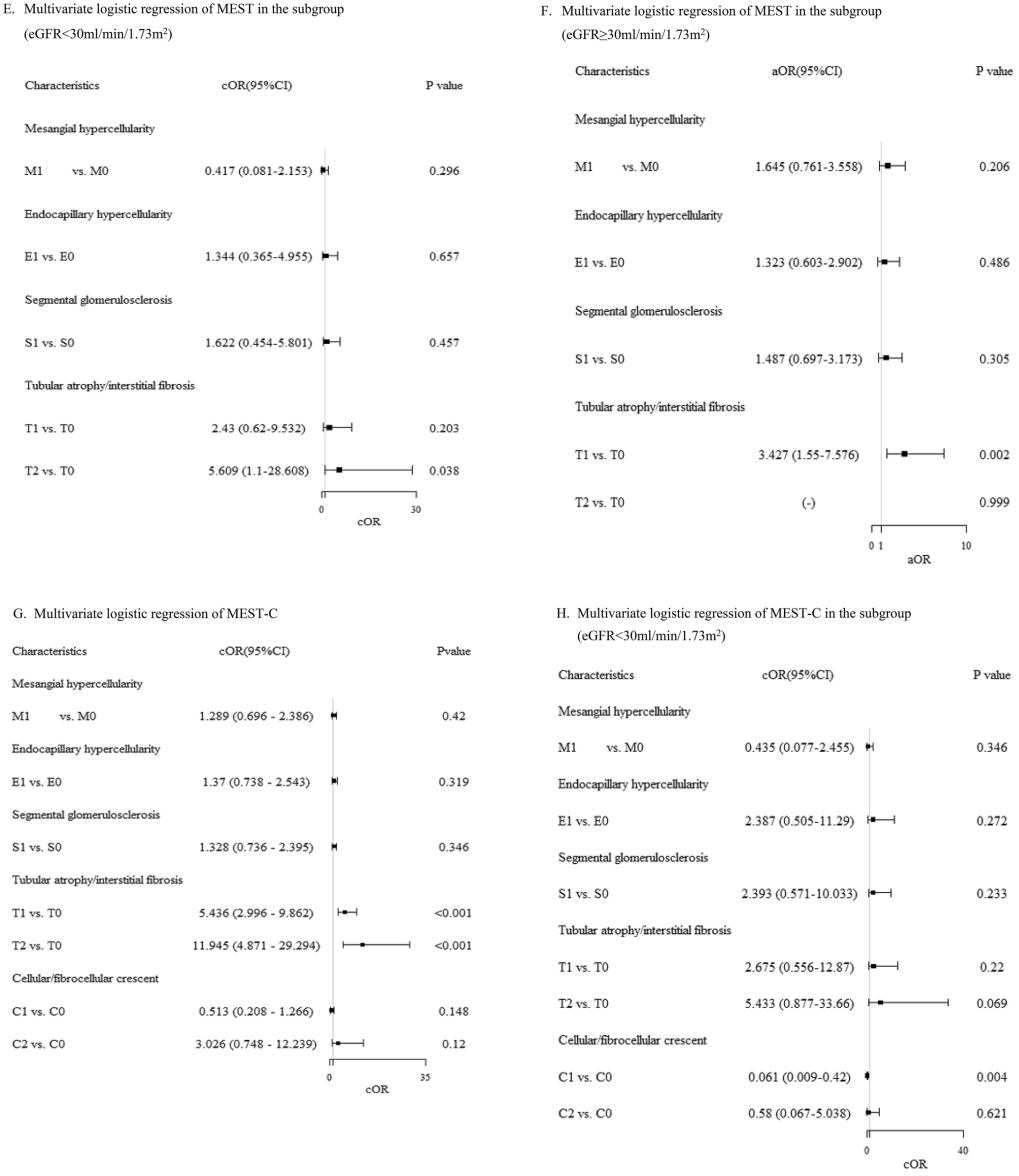

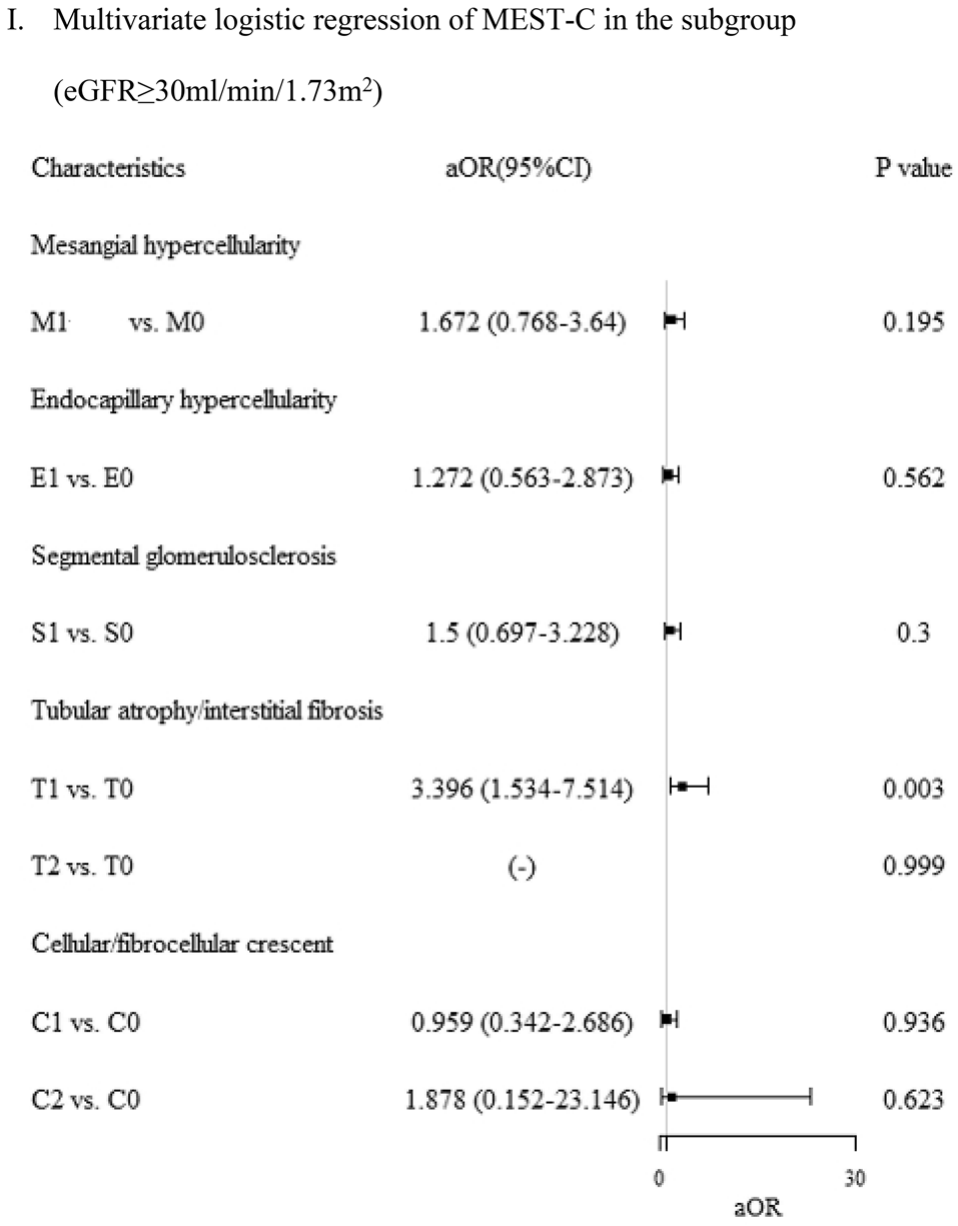
Adjusted odds ratio for each all pathological variables comparison. (**A**) Univariate logistic regression of individual M, E, S, T, and C. (**B**) Univariate logistic regression of individual M, E, S, T, and C in the subgroup (eGFR < 30 ml/min/1.732 m^2^). (**C**) Univariate logistic regression of individual M, E, S, T, and C in the subgroup (eGFR ≥ 30 ml/min/1.73 m^2^). (**D**) Multivariate logistic regression of MEST. (**E**) Multivariate logistic regression of MEST in the subgroup (eGFR < 30 ml/min/1.73 m^2^). (**F**) Multivariate logistic regression of MEST in the subgroup (eGFR ≥ 30 ml/min/1.73 m^2^). (**G**) Multivariate logistic regression of MEST-C. (**H**) Multivariate logistic regression of MEST-C in the subgroup (eGFR < 30 ml/min/1.73 m^2^). (**I**) Multivariate logistic regression of MEST-C in the subgroup (eGFR ≥ 30 ml/min/1.73 m^2^).

In univariable logistic analysis (Fig. 2A), all individual M, E, S, T, and C variables correlated with worse outcomes (except C1 vs. C0, p = 0.418). Highest OR was T2 vs. T0 (14.7, 95%CI 6.132–35.029), followed by T1 vs. T0 (6.0, 95% CI 3.40–10.67), C2 vs. C0 (4.2, 95% CI 1.21–14.77). Moving to multivariable logistic regression for MEST (Fig. 2D), only T1 vs. T0 and T2 vs. T0 significantly associated with worse outcomes (5.3, 95% CI 2.96–9.58 and 12.0, 95% CI 5.00–29.61, both p < 0.001). In another analysis of multivariable logistic regression of MEST-C (Fig. 2G), T1 vs. T0 and T2 vs. T0 were significant: 5.4, 95% CI 3.0–9.86 and 12.0, 95% CI 4.87–29.29, both p < 0.001. In Supplementary Fig. 1A,B, Hosmer–Lemeshow test assessed MEST and MEST-C models. Both p values were > 0.05, and points aligned with red line. Models matched data well, indicating good fit. Similarly, in Supplementary Table 3 (univariable/multivariable Cox-proportional hazard analysis), T1 vs. T0 and T2 vs. T0 were associated with worse renal outcome: 4.32 (2.72–6.85) and 6.28 (3.55–11.11), respectively (both p < 0.001).

In univariable logistic analysis of individual M, E, S, T, and C for eGFR < 30 ml/min/1.732 m^2^ (Fig. 2B), T2 vs. T0 is associated with worse renal function (4.72, 1.06–20.96) and C1 vs. C0 is associated with better renal outcome (0.136, 0.03–0.65). In univariable logistic analysis of individual M, E, S, T, and C for eGFR ≥ 30 ml/min/1.732 m^2^ (Fig. 2C), M1 vs. M0 and T1 vs. T0 linked to worse renal function (2.06, 1.02–4.18 and 3.7, 1.71–8.13, respectively). In multivariable logistic analysis of MEST for eGFR < 30.0 ml/min/1.73 m^2^ subgroup (Fig. 2E), T2 vs. T0 linked to worse outcome (5.61, 1.10–28.61). In eGFR ≥ 30.0 ml/min/1.73 m^2^ subgroup (Fig. 2F), only T1 vs. T2 associated with worse outcome (3.4, 1.55–7.58). For multivariable logistic regression of MEST-C in eGFR < 30.0 ml/min/1.73 m^2^ subgroup (Fig. 2H), C1 vs. C0 linked to better outcome (0.06, 0.01–0.42). In eGFR ≥ 30.0 ml/min/1.73 m^2^ subgroup (Fig. 2I), T1 vs. T0 associated with worse outcome (3.4, 1.53–7.51). Supplementary Fig. 1C–F [subgroups for model 1 (MEST) and model 2 (MEST-C)] showed Hosmer–Lemeshow goodness of fit test p values above 0.05, aligning with red line. Model estimates matched data well, indicating good fit.

### Predictive power for composite renal outcomes based on pathological variables of MEST-C

**Figure 3:**
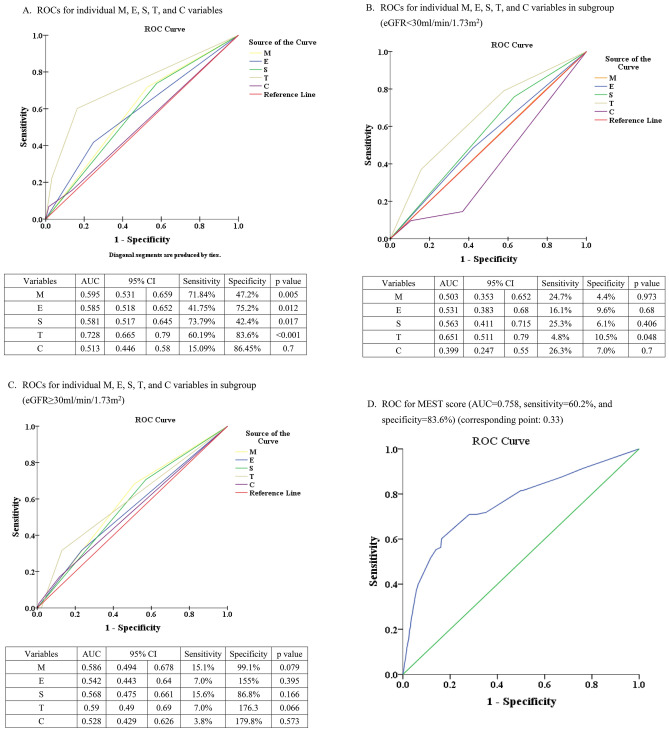

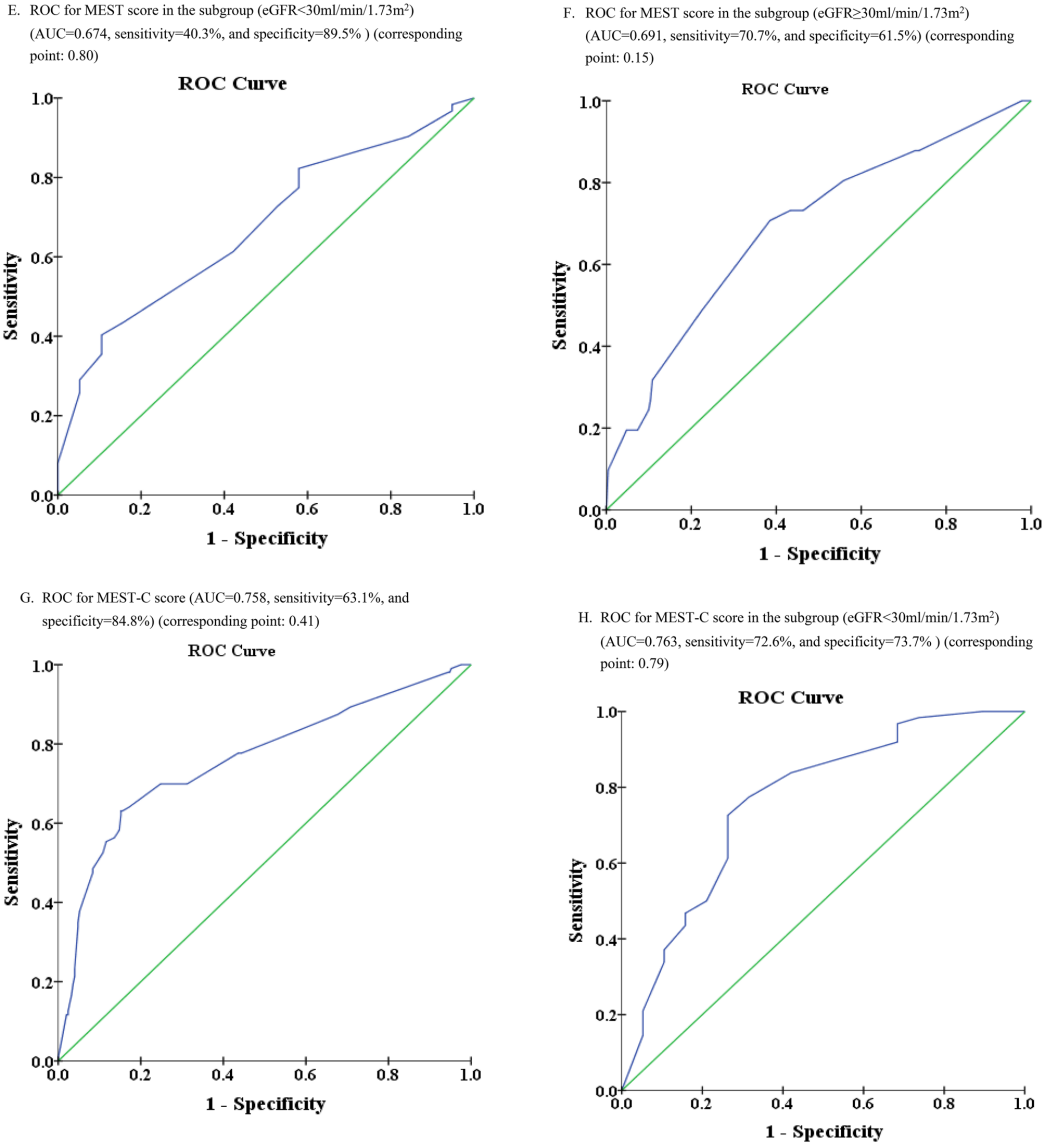

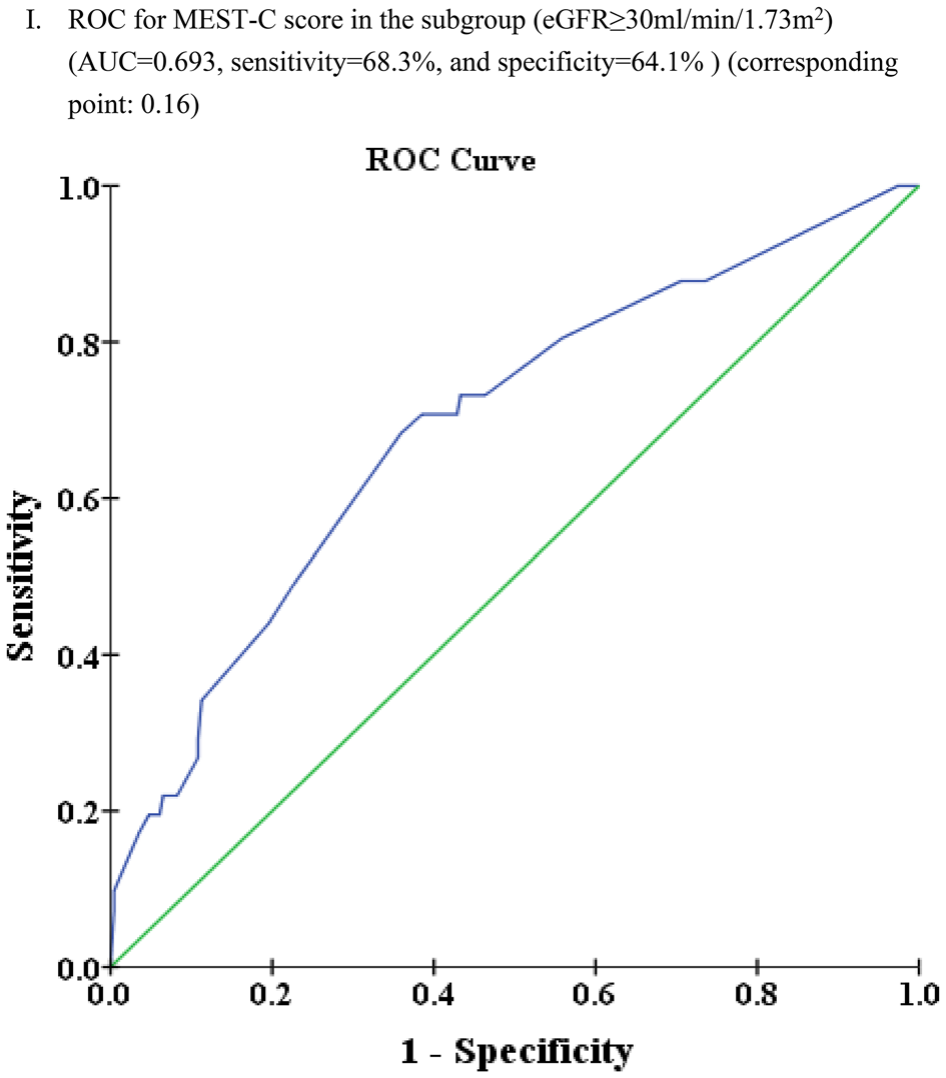
Prediction of composite renal outcome compared by different AUCs of ROC. (**A**) ROCs for individual M, E, S, T, and C variables. (**B**) ROCs for individual M, E, S, T, and C variables in subgroup (eGFR < 30.0 ml/min/1.73 m^2^). (**C**) ROCs for individual M, E, S, T, and C variables in subgroup (eGFR ≥ 30.0 ml/min/1.73 m^2^). (**D**) ROC for MEST score (AUC = 0.76, sensitivity = 60.2%, and specificity = 83.6%). (**E**) ROC for MEST score in the subgroup (eGFR < 30.0 ml/min/1.73 m^2^) (AUC = 0.67, sensitivity = 40.3%, and specificity = 89.5%). (**F**) ROC for MEST score in the subgroup (eGFR ≥ 30.0 ml/min/1.73 m^2^) (AUC = 0.69, sensitivity = 70.7%, and specificity = 61.5%). (**G**) ROC for MEST-C score (AUC = 0.76, sensitivity = 63.1%, and specificity = 84.8%). (**H**) ROC for MEST-C score in the subgroup (eGFR < 30.0 ml/min/1.73 m^2^) (AUC = 0.76, sensitivity = 72.6%, and specificity = 73.7%). (**I**) ROC for MEST-C score in the subgroup (eGFR ≥ 30.0 ml/min/1.73 m^2^) (AUC = 0.69, sensitivity = 68.3%, and specificity = 64.1%).

Predictive power shown in Fig. 3A for individual M, E, S, T, C variables, highest power was in T score (AUC 0.73, sensitivity 60.2%, specificity 83.6%). AUCs for other variables were less than 0.6, indicating lower predictiveness. In Fig. 3D, the predictive power of MEST score was high: AUC 0.76, sensitivity 60.2%, specificity 83.6%. After adding C score to MEST-C (Fig. 3G), AUC remained at 0.76, with slight sensitivity increase (63.0%) and specificity increase (84.8%). Comparing Fig. 3G,D, C lesion did not enhance predictive power (same AUC 0.76) (p = 0.8792 by Delong test), but slightly increased sensitivity (60.2–63.1%) and specificity (83.5–84.8%).

In subgroup-specific ROC analysis for eGFR < 30.0 ml/min/1.73 m^2^ (Fig. 3B), T lesion had highest predictive power (AUC 0.65). Interestingly, C lesion predicted better outcome in this subgroup. In alternate subgroup (eGFR ≥ 30.0 ml/min/1.73 m^2^) (Fig. 3C), none of the lesions had strong predictive ability, though T lesion still had highest AUC.

For MEST predictions, abilities were comparable in both eGFR groups (AUC 0.67 for eGFR < 30.0 in Fig. 3E, and 0.69 for eGFR ≥ 30.0 ml/min/1.73 m^2^ in Fig. 3F). However, with MEST-C, AUC was higher (0.763) in eGFR < 30.0 group (Fig. 3H) than eGFR ≥ 30.0 ml/min/1.73 m^2^ group (Fig. 3I) with AUC 0.69. To summarize, C lesion numerically increased the predictive power notably within the eGFR < 30.0 ml/min/1.73 m^2^ group (Fig. 3H vs. E) (p = 0.1097 by Delong test). Conversely, in eGFR ≥ 30.0 ml/min/1.73 m^2^ group (Fig. 3I vs. F), C lesion did not contribute more predictive power (p = 0.8192 by Delong test).

## Discussion

All significant ORs and AUC values have been summarized and are presented in Supplementary Tables 1 and 2. We observed that the most consistently impactful variables were T lesions, followed by C lesions. M lesions were associated with a worse renal outcome in univariable analysis and within the subgroup with eGFR ≥ 30.0 ml/min/1.73 m^2^. On the other hand, E and S lesions showed an association with worse renal outcomes only in univariable analysis. These findings warrant further discussion. Before further discussion, we should keep in mind that predicting the followed-up renal outcome involves considering several factors, including baseline eGFR, the severity and chronicity of pathological lesions, lesion characteristics, the indication, and the treatment protocol (specifically, the aggressiveness of immunosuppression), along with the treatment response. Additionally, it is important to recognize that the variability in reported study results is influenced by the reproducibility of lesion assessments, whether they are subjective or objective.

The E lesion did not exhibit an association with a worse renal outcome in multivariable analysis and lacked predictive power. This outcome aligns with the findings of the majority of studies. In a review article^12^, the results were consistent across the majority of studies (16 out of 19). However, it is worth noting that two studies suggested a possible influence from treatment bias^22,23^. Furthermore, a majority of studies have also reported poor reproducibility of the E lesion^11^, which introduces certain limitations when discussing the significance of the E lesion.

The S lesion only shows an association in univariable analysis and lacks predictive power, which is consistent with a previous report^12^. Only a subset of studies (8 out of 19) supports the predictive capacity of the S lesion. There are several reasons for this. The S lesion is linked to podocyte injury^24^ and can indicate higher proteinuria levels. Notably, we did not include proteinuria as part of our renal outcome assessment. Furthermore, the S lesion might respond favorably to immunosuppression, potentially introducing treatment bias when predicting worse renal outcomes Lastly, it is worth noting that the reproducibility of the S lesion is rated as moderate to poor^11^. Collectively, these factors contribute to the lack of predictive power of the S lesion.

The T lesions consistently displayed a strong association with worse renal outcomes across all conditions, except for the MEST score in cases with an eGFR ≥ 30.0 ml/min/1.73 m^2^. Furthermore, in the multivariable analysis, only T1 and T2 exhibited significant associations. Regarding the predictive power for worse renal outcomes, the T lesion exhibited the highest AUC (0.73), followed by 0.65 for an eGFR of < 30.0 ml/min/1.73 m^2^ and 0.59 for an eGFR ≥ 30.0 ml/min/1.73 m^2^. These findings are consistent with previous studies indicating that the T lesion consistently presents across various studies^8,11,12,25^ that the T score demonstrated the most robust predictive capability for renal outcomes. In the initial meta-analysis conducted in 2013 (16 retrospective cohort studies, 3893 patients)^8^, the hazard ratios (HRs) for kidney failure were notably elevated for T1/T2 (3.2, 95% CI 1.8–5.6). Correspondingly, in the most recent systematic review (99 relevant studies up to September 23, 2022)^11^, the Oxford element most frequently linked with outcomes was T (73 out of 125, 58%). Several reasons can elucidate the value of the T lesion. Firstly, the reproducibility of the T lesion is good^11^ among all five variables. The T score is relatively less subjective compared to the other four variables in our analysis, contributing to consistent findings across studies. Secondly, the T lesion indicates chronic changes, and clinicians often opt for less aggressive immunosuppressant approaches for T lesions. Additionally, these chronic changes are generally resistant to complete reversal through treatment. Therefore, the T lesion is less susceptible to treatment bias. Thirdly, an early study published in The Lancet concluded that in cases of persistent glomerular nephritis, alterations in tubules have a more pronounced impact on GFR than changes in glomeruli^26^. Based on the aforementioned reasons, the baseline T lesion independently factors into long-term renal outcomes regardless of treatment and baseline eGFR status.

Shifting the focus to the C lesions, initially, C1 and C2 were associated with lower and much lower baseline eGFR, respectively (Table 2). Then, in Supplementary Table 1, after a long-term follow-up, C2 displayed an association with worse renal outcomes in the univariable analysis; however, this significance did not hold true in the multivariable analysis. In contrast, within the subgroup analysis (eGFR < 30.0 ml/min/1.73 m^2^), the C1 lesion was linked to improved renal outcomes in both univariable and multivariable analyses using the MEST-C model. Regarding the merged lesion score (Supplementary Table 2), the C lesion did not contribute additional predictive power during the transition from MEST to MEST-C (the same AUC value of 0.758). Our findings are in line with other studies that also demonstrated no association between C lesions and worse renal outcomes^27–30^.

When discussing the C lesion, it is crucial to consider all associated factors, including baseline eGFR, lesion severity and chronicity, various characteristics of the lesion, the indication, treatment protocol, as well as the treatment response. Initially, baseline eGFR is markedly lower in cases with C1 lesions and significantly lower in cases with C2 lesions (Table 2). Furthermore, the severity of the C2 lesion surpasses that of C1, and it also exhibits a more advanced degree of chronicity. In our treatment protocol, we adopt an aggressive approach for all cases with C lesions, involving plasmapheresis and methylprednisolone pulse therapy. However, there is a possibility that the response to treatment could be more favorable for C1 lesions compared to C2 lesions due to the lesser presence of chronic changes.

The treatment with immunosuppressants for C lesions deserves particular attention in this discussion. The utilization of immunosuppression post-biopsy has frequently been associated with C and E lesions^11^. In a combined cohort study^12^, the C lesion was independently predictive of a worse renal outcome (HR 1.37, 95% CI 1.07–1.75) when not treated with immunosuppressants. Numerous studies also reinforce the notion that the C1 lesion identifies individuals at risk of an unfavorable renal outcome if not subjected to immunosuppressive treatment^5,10,31–35^.

In our investigation, when contrasting the C and T lesions, it is essential to recognize that even though the T and C lesions are characterized by lower baseline eGFR (Table 2), the C lesion is not consistently associated with a worse renal outcome. This shift in association can be attributed to various factors, notably the implementation of aggressive treatment measures, the prevalence of acute lesions displaying fewer chronic changes, and a more positive response to immunosuppressive intervention. Consequently, the C1 lesion ultimately aligns with an improved renal outcome.

When comparing C lesion between different subgroups, the improved renal outcome by C1 in eGFR < 30.0 not in eGFR ≥ 30.0 ml/min/1.73 m^2^ maybe due to much aggressive treatment in eGFR < 30.0 ml/min/1.73 m^2^ group (Supplementary Table 1). The more aggressive treatment can also explain the predictive power of C in eGFR < 30.0 but not in eGFR ≥ 30.0 ml/min/1.73 m^2^ group (Supplementary Table 2).

Comparing C1 (cellular) and C2 (fibrocellular) lesions reveals that C2 lesions are linked to a lower baseline eGFR, more pronounced chronic changes, and a comparatively weaker response to immunosuppressants. As a result, the C1 lesion can transition from its initially associated worse renal outcome to a more favorable future renal status. Conversely, for the C2 lesion, there exists the potential for it to shift from an initially worse renal outcome association to an improved outcome (albeit to a lesser extent than C1). This finding aligns with a previous study^9^ that similarly indicates C2 lesions continue to predict worse renal outcomes.

Regarding the predictive power (Supplementary Table 2), the C lesion did not provide additional power when transitioning from MEST (AUC of 0.76, Fig. 3D) to MEST-C (AUC of 0.76, Fig. 3H). However, the MEST-C model appears to predict worse outcomes more effectively when the eGFR is < 30.0 ml/min/1.73 m^2^ (AUC of 0.76 in Fig. 3H compared to AUC of 0.69 in Fig. 3I). This phenomenon could potentially be attributed to the lack of differentiation between C1 and C2 lesions in this analysis. As we established in our earlier discussion, C1 and C2 possess distinct characteristics. In Fig. 3H, the impact of significantly lower baseline eGFR appears to be particularly influential.

There are some limitations to this study. The first one is that it is a single-center study conducted in our country. However, within our institute, we have recorded the highest number of renal biopsies in Taiwan to date^2,13^, amassing more than 8000 cases of renal biopsies. Therefore, we believe that our institute can be considered representative of the Taiwanese population. Secondly, we did not incorporate treatment factors into this cohort study, and we only included cases of first-time biopsies and without immunosuppression. As a result, we cannot determine the effect of immunosuppression on C lesion. In the future, we will investigate the effect of treatment by enrolling patients who have undergone repeated renal biopsies.

## Conclusion

We discovered that the T lesion consistently correlates with worse renal outcomes across all groups and baseline statuses. When we incorporated the C lesion into the transition from MEST to MEST-C, we did not observe an increase in predictive power. Notably, the C1 lesion is linked to improved renal outcomes in the eGFR < 30.0 ml/min/1.73 m^2^ subgroup, possibly due to treatment effects. From our findings, we conclude that the T lesion can be employed to predict renal outcomes, while the C1 lesion requires careful consideration of various factors, particularly the influence of treatment bias.

### Supplementary Information

Supplementary Information.

## Data Availability

All data generated or analyzed during this study are included in this published article and its supplementary information files.
