# Supplementary material for: Validating the association of Oxford classification and renal function deterioration among Taiwanese individuals with Immunoglobulin A nephropathy
Source: Sci Rep. 2023 Dec 11;13:21904. doi: 10.1038/s41598-023-49331-7 (PMC10713632; doi:10.1038/s41598-023-49331-7)
Supplement: Supplementary file 1 — Supplementary Information. [file 41598_2023_49331_MOESM1_ESM.pdf]

Supplementary data

Table 1. Summary of significance among all univariable and multivariable logistic regression analyses, including subgroup analysis for all eGFR values

Table 2. Summary of AUCs from ROC analysis, including subgroup analysis for all cases

Table 3. Cox-proportional hazard analysis for outcome.

Figure 1. Hosmer-Lemeshow goodness-of-fit for logistic regression.

A. Model 1: MEST
B. Model 1: MEST-C
C. Model 1 in the subgroup (eGFR<30ml/min/1.732m2): MEST
D. Model 1 in the subgroup (eGFR≥30ml/min/1.732m2): MEST
E. Model 2 in the subgroup (eGFR<30ml/min/1.732m2): MEST-C
F. Model 2 in the subgroup (eGFR≥30ml/min/1.732m2): MEST-C

Table 1. Summary of significance among all univariable and multivariable logistic regression analyses, including subgroup analysis for all eGFR values

Univariable	M, E, S, T, and C	All eGFR: M, E1, S1, T1, T2, and C2	GFR<30	T2, C1*
			eGFR≥30	M, T1
Multivariable	MEST	All eGFR: T1, T2	GFR<30	T2
			eGFR≥30	
	MEST-C	All eGFR: T1, T2	GFR<30	C1*, T2 (p=0.069)
			eGFR≥30	T1

All are significantly increased risk, only *significantly lower risk

Table 2. Summary of AUCs from ROC analysis, including subgroup analysis for all cases

Individual lesions	M, E, S, T, and C	T (0.728), M (0.595), E (0.585), S (0.581)	GFR<30	T (0.651), C*
			eGFR≥30	T (0.59) (p=0.066)
Merged lesions	MEST	0.758	GFR<30	0.674
			eGFR≥30	0.691
	MEST-C	0.758	GFR<30	0.763
			eGFR≥30	0.693

*ROC curve below diagonal line, but without significance

Table 3. Cox-proportional hazard analysis for outcome.

Characteristics	cHR (95%CI)	p-value	aHR (95%CI)	P value
Demographic data				
Age	1.01 (0.996 - 1.024)	0.176		
Gender (female vs. male)	1.151 (0.793 - 1.67)	0.459		
Pathological data				
Mesangial hypercellularity				
M1 vs. M0	1.831 (1.188 - 2.822)	0.006		
Endocapillary hypercellularity				
E1 vs. E0	2.41 (1.615 - 3.596)	<0.001		
Segmental glomerulosclerosis				
S1 vs. S0	1.581 (1.015 - 2.461)	0.043		
Tubular atrophy/interstitial fibrosis		0.011		
T1 vs. T0	4.686 (2.971 - 7.389)	<0.001	4.317 (2.719 - 6.854)	<0.001
T2 vs. T0	8.406 (4.959 - 14.25)	<0.001	6.281 (3.55 - 11.111)	<0.001
Cellular/fibrocellular crescent		0.879		
C1 vs. C0	1.303 (0.652 - 2.604)	0.454		
C2 vs. C0	5.092 (2.181 - 11.888)	<0.001		
Comorbidity				
Diabetes mellitus	1.375 (0.853 - 2.217)	0.191		
Coronary arterial disease	1.258 (0.656 - 2.412)	0.49		
Arrythmia	1.622 (0.596 - 4.415)	0.344		
Congestive heart failure	18.196 (2.557 - 129.488)	0.004		
Laboratory data				
Serum albumin	0.374 (0.275 - 0.508)	<0.001	0.434 (0.302 - 0.624)	<0.001
UPCR	1.000126 (1.000089 - 1.000162)	<0.001		
Fasting blood glucose	1.004 (1 - 1.009)	0.064		

Glycated hemoglobin	1.076 (0.831 - 1.393)	0.578		
Low-density lipoprotein	1.003 (1 - 1.007)	0.033		
Uric acid	1.078 (1.034 - 1.124)	<0.001		
Serum IgA/C3 ratio	1.041 (0.935 - 1.16)	0.465		
Medication				
Renin–angiotensin system inhibitors	1.547 (0.805 - 2.974)	0.19		
Pentoxifylline	0.785 (0.538 - 1.145)	0.209		
Dipyridamole	0.447 (0.109 - 1.833)	0.263		
Statin	1.678 (1.133 - 2.486)	0.01		

* Variables demonstrating a p-value of 0.2 or less in the univariate analysis were chosen to participate in a backward selection algorithm, aiming to derive a more concise multivariable regression model. The proportional hazards assumption was assessed using scaled Schoenfeld residuals. Hazard ratios and their corresponding 95% confidence intervals (CI) were reported. The selected variables included age, M1 vs. M0, E1 vs. E0, S1 vs. S0, tubular atrophy/interstitial fibrosis, T1 vs. T0, T2 vs. T0, C2 vs. C0, diabetes mellitus, congestive heart failure, serum albumin, UPCR, fasting blood glucose, LDL, uric acid, renin-angiotensin system inhibitors, and statins.

Figure 1. Hosmer-Lemeshow goodness-of-fit for logistic regression.

A. Model 1: MEST

Contingency Table for Hosmer and Lemeshow Test						
		outcome = 0		outcome = 1		Total
		Observed	Expected	Observed	Expected	
Step 1	1	60	60.678	9	8.322	69
	2	23	23.119	4	3.881	27
	3	40	39.083	6	6.917	46
	4	39	40.304	10	8.696	49
	5	18	15.267	1	3.733	19
	6	29	30.548	11	9.452	40
	7	25	22.87	21	23.13	46
	8	10	13.077	25	21.923	35
	9	6	5.054	16	16.946	22
Hosmer and Lemeshow Test						
Step	Chi-square	df	Sig.			
1	5.051	7	0.654			

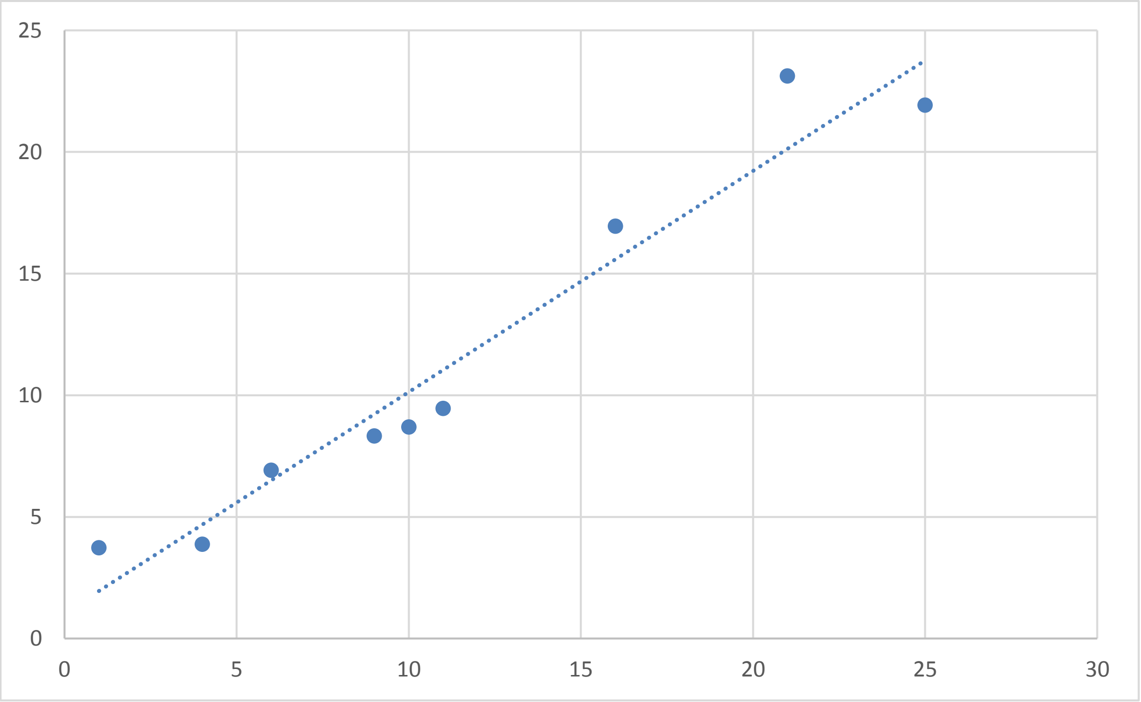

B. Model 2: MEST-C

Contingency Table for Hosmer and Lemeshow Test						
		outcome = 0		outcome = 1		Total
		Observed	Expected	Observed	Expected	
Step 1	1	59	59.792	9	8.208	68
	2	24	23.997	4	4.003	28
	3	35	34.906	6	6.094	41
	4	38	38.027	8	7.973	46
	5	24	21.781	3	5.219	27
	6	27	26.817	8	8.183	35
	7	14	15.434	13	11.566	27
	8	15	13.336	14	15.664	29
	9	14	15.909	38	36.091	52
Hosmer and Lemeshow Test						
Step	Chi-square	df	Sig.			
1	2.289	7	0.942			

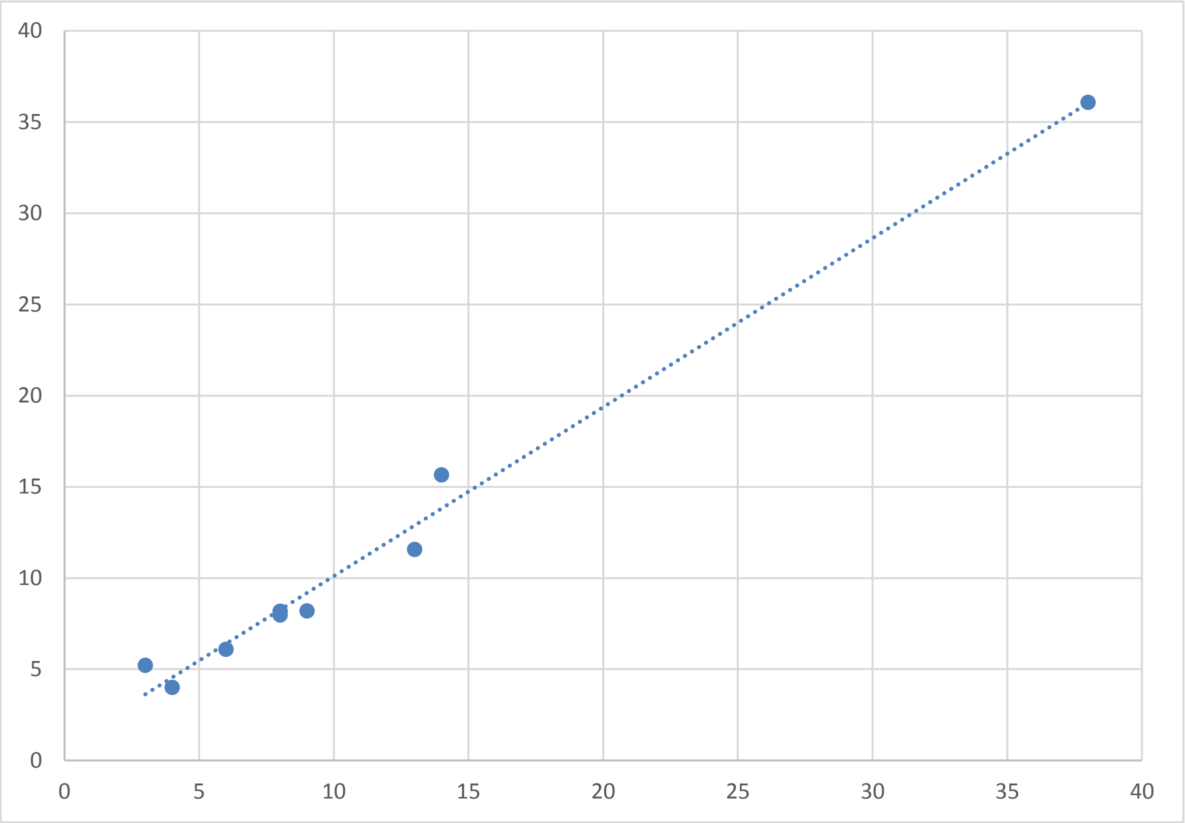

C. Model 1 in the subgroup (eGFR<30ml/min/1.732m^2): MEST

Contingency Table for Hosmer and Lemeshow Test						
		outcome = .00		outcome = 1.00		Total
		Observed	Expected	Observed	Expected	
Step 1	1	3	3.984	6	5.016	9
	2	4	2.955	4	5.045	8
	3	2	2.696	7	6.304	9
	4	2	2.365	7	6.635	9
	5	5	3.353	11	12.647	16
	6	1	1.147	5	4.853	6
	7	1	0.928	6	6.072	7
	8	1	1.237	11	10.763	12
	9	0	0.336	5	4.664	5
Hosmer and Lemeshow Test						
Step	Chi-square	df	Sig.			
1	2.819	7	0.901			

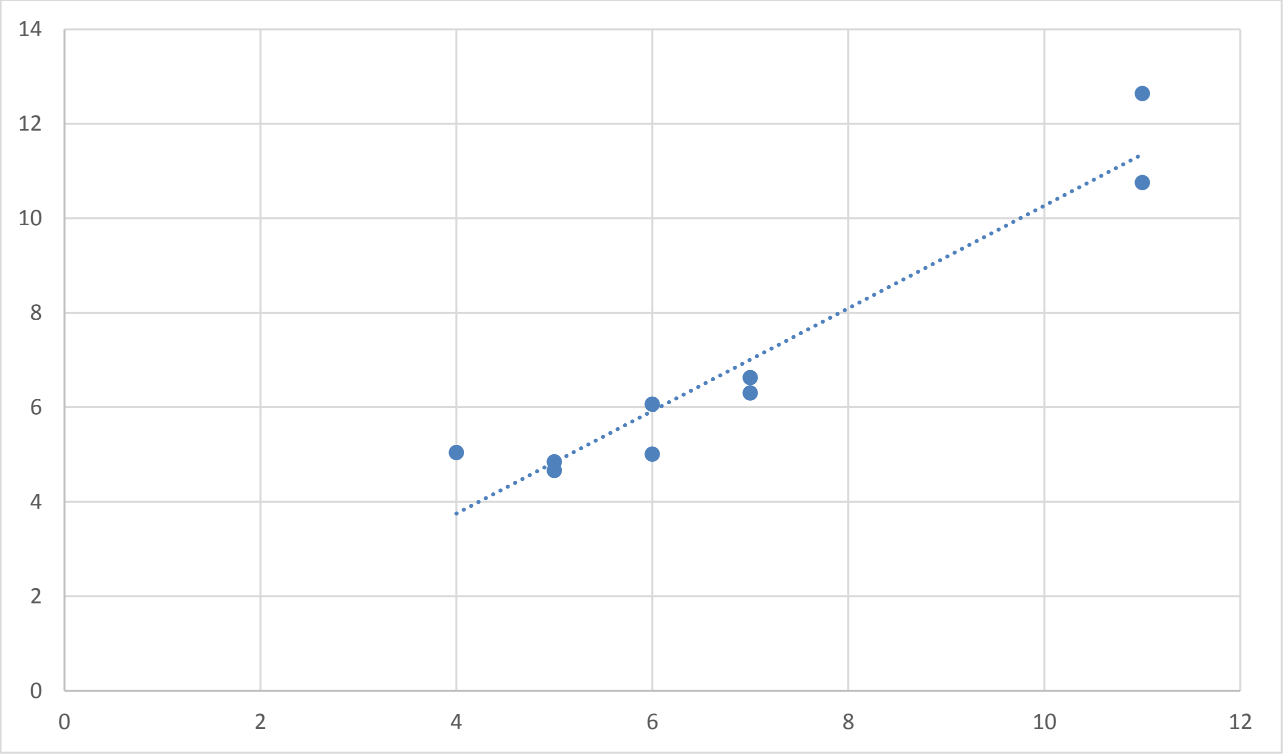

D. Model 1 in the subgroup (eGFR≥30ml/min/1.732m2): MEST

		outcome = .00		outcome = 1.00		Total
		Observed	Expected	Observed	Expected	
Step 1	1	5	5	0	0	5
	2	56	56.702	5	4.298	61
	3	41	39.564	3	4.436	44
	4	22	22.228	3	2.772	25
	5	18	16.393	1	2.607	19
	6	37	38.806	9	7.194	46
	7	27	27.305	7	6.695	34
	8	25	25	13	13	38
Hosmer and Lemeshow Test						
Step	Chi-square	df	Sig.			
1	2.364	6	0.883			

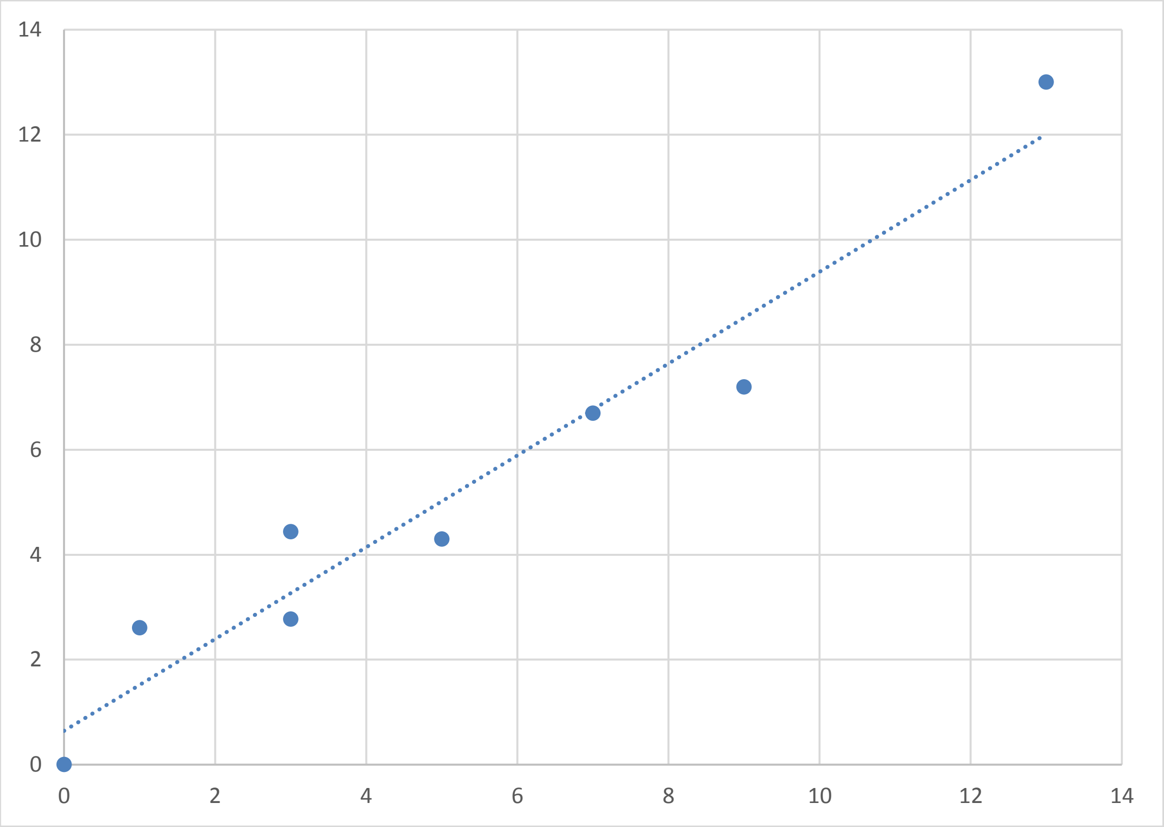

E. Model 2 in the subgroup (eGFR<30ml/min/1.732m^2): MEST-C

Contingency Table for Hosmer and Lemeshow Test							
		outcome = .00		outcome = 1.00		Total	
		Observed	Expected	Observed	Expected		
Step 1	1	6	5.315	2	2.685	8	
	2	4	4.624	7	6.376	11	
	3	3	2.341	5	5.659	8	
	4	1	1.695	7	6.305	8	
	5	1	2.129	10	8.871	11	
	6	2	1.186	8	8.814	10	
	7	1	1.011	10	9.989	11	
	8	0	0.228	4	3.772	4	
	9	1	0.471	9	9.529	10	
Hosmer and Lemeshow Test							
Step	Chi-square	df	Sig.				
1	3.272	7	0.859				

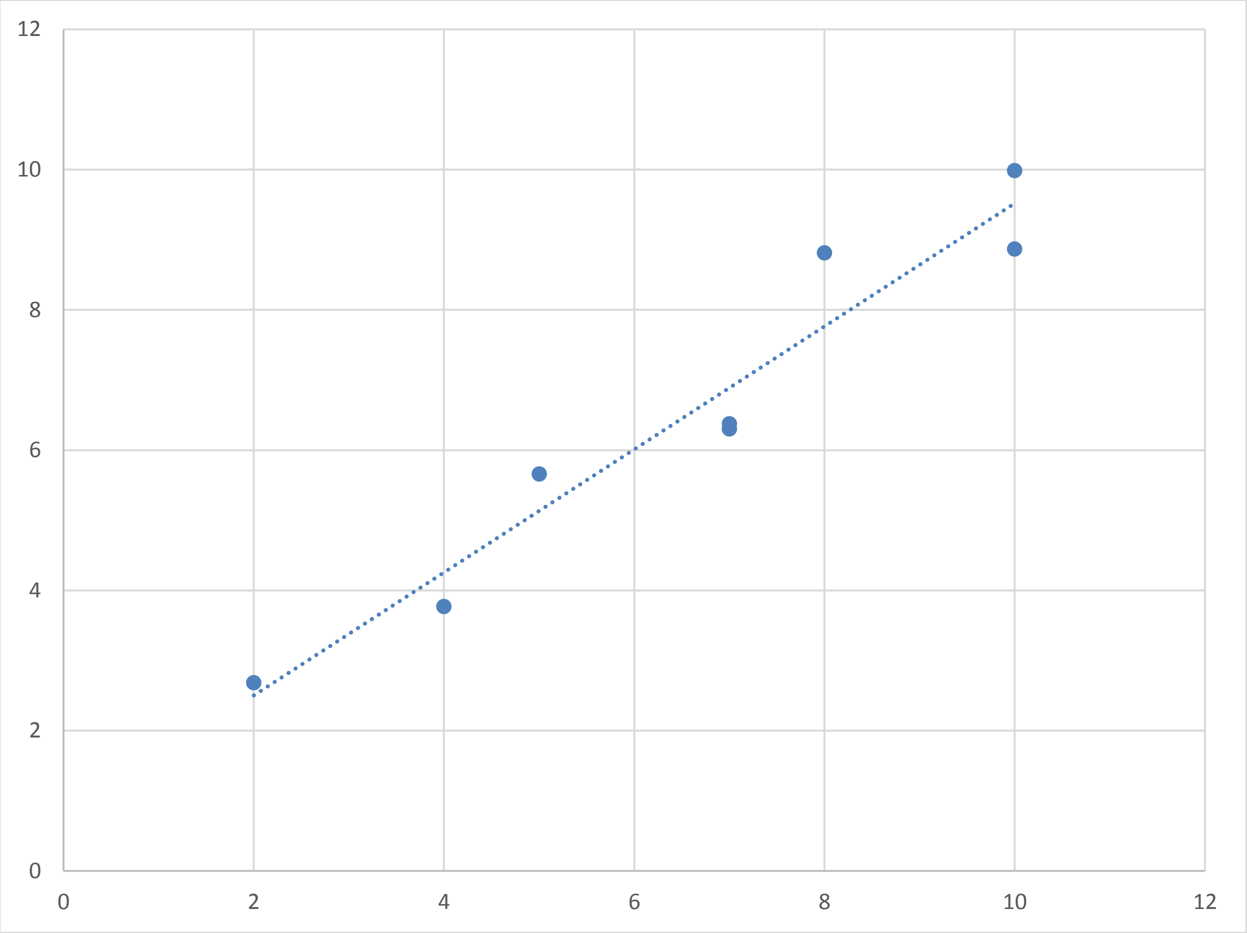

F. Model 2 in the subgroup (eGFR≥30ml/min/1.732m^2): MEST-C

		outcome = .00		outcome = 1.00		Total
		Observed	Expected	Observed	Expected	
Step 1	1	6	5.933	0	0.067	6
	2	55	55.804	5	4.196	60
	3	41	39.587	3	4.413	44
	4	23	23.087	3	2.913	26
	5	23	21.53	2	3.47	25
	6	31	32.813	8	6.187	39
	7	26	25.867	6	6.133	32
	8	18	17.516	7	7.484	25
	9	8	8.862	7	6.138	15
	Hosmer and Lemeshow Test					
	Step	Chi-square	df	Sig.		
	1	2.347	7	0.938		

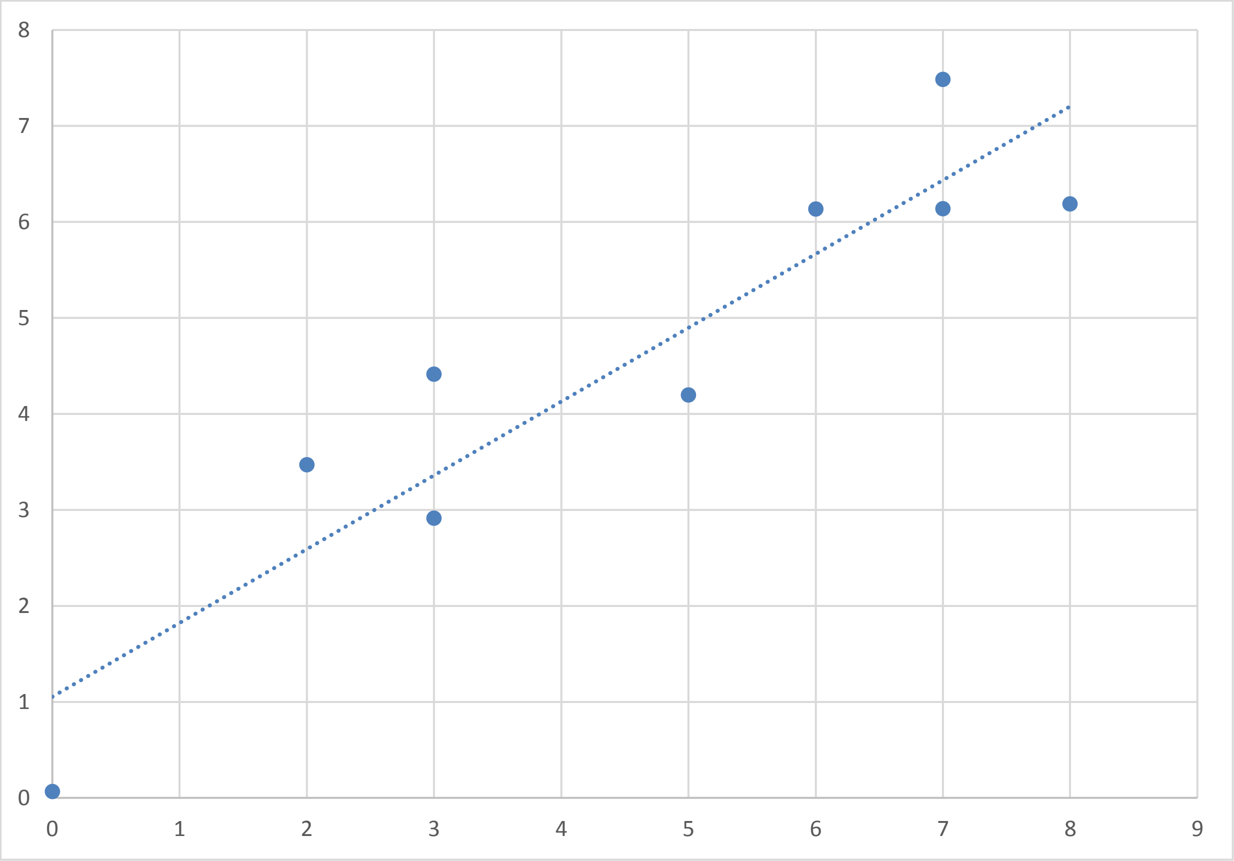

List of abbreviations

- area under the curve (AUC)
- body mass index (BMI)
- complement 3 (C3),
- estimated glomerular filtration rate (eGFR)
- fasting blood sugar (FBS)
- glycated hemoglobin (HbA1c)
- high-density lipoprotein (HDL)
- Immunoglobulin A nephropathy (IgAN)
- Kidney Disease: Improving Global Outcomes (KDIGO)
- low-density lipoprotein (LDL)
- MEST-C: mesangial hypercellularity (M), endocapillary hypercellularity (E), segmental glomerulosclerosis (S), tubular atrophy/interstitial fibrosis (T), and crescents (C)
- Odds ratio (OR)
- receiver operating characteristic curves (ROC curves)
- serum creatinine (SCr)
- total cholesterol (TC)
- urine protein-creatinine ratio (UPCR)

uric acid (UA)
